# A Glycoside Hydrolase Family 62 A-L-Arabinofuranosidase from *Trichoderma Reesei* and Its Applicable Potential during Mashing

**DOI:** 10.3390/foods9030356

**Published:** 2020-03-19

**Authors:** Junyong Sun, Feng Xu, Jian Lu

**Affiliations:** 1 The Key Laboratory of Industrial Biotechnology, Ministry of Education, Jiangnan University, Wuxi 214122, Jiangsu, China; jysun@jiangnan.edu.cn; 2 National Engineering Laboratory for Cereal Fermentation Technology, Jiangnan University, Wuxi 214122, Jiangsu, China; 3 School of Biotechnology, Jiangnan University, Wuxi 214122, Jiangsu, China; 4 Jiangsu Provincial Research Center for Bioactive Product Processing Technology, Jiangnan University, Wuxi 214122, Jiangsu, China; 5 Wuxi Newway Biotechnology Co. Ltd., 100 Konggang Road, Wuxi 214122, Jiangsu, China; xufeng618@163.com

**Keywords:** α-L-arabinofuranosidase, *Trichoderma reesei*, arabinoxylan, malted barley, mashing

## Abstract

Arabinoxylan is the second most abundant component in the endosperm cell wall of barley and it has been shown to have negative effects on the viscosity and filtration rate of wort and beer. In this study, a glycoside hydrolase (GH) family 62 α-L-arabinofuranosidase (AFase), termed as TrAbf62A, was purified from the culture filtrate of *Trichoderma reesei* CICC 41495 by a combined chromatographic method. The preferred substrates of the purified TrAbf62A were soluble, highly substituted arabinoxylan oligosaccharides and polymers, similar to the type found in barley grain. TrAbf62A exhibited activity towards oligomeric and polymeric arabinoxylans, as well as colorimetric arabinose-based substrates, thus meeting the criteria to be classified as a type B AFase. TrAbf62A released mainly arabinose and xylose from soluble wheat arabinoxylan, thus indicating a dual lytic enzyme activity. Supplementation of TrAbf62A during mashing, with a loading of 12 mU/g malt, resulted in a 36.3% decrease in arabinoxylan polymer content, a 5.6% reduction in viscosity, and finally, a 22.1% increase in filtration rate. These results revealed that TrAbf62A has a high potential value in improving lautering performance during mashing.

## 1. Introduction

The separation of sweet wort from the mash is usually the most problematic step in the brewhouse. Poor filtration efficiency has been mainly attributed to β-glucan, which is the major non-starch polysaccharide in the cell wall of barley malt [1,2]. The main focus of improving the lautering performance of wort has been on β-glucan content and β-glucanase activity. However, when some domestic barley varieties and subsequently produced malts were used for beer brewing, even though the β-glucan content was low (<100 mg/L), the lautering performance was still poor. Latest researches have shown that arabinoxylan polymer may be involved in this phenomenon [3,4,5,6,7].

Arabinoxylan is the second most abundant component in the endosperm cell wall of barley [8]. Arabinoxylan consists of a β-1,4-D-xylopyranosyl backbone, substituted with arabinofuranosyl residues at C-(*O*)-2 or C-(*O*)-3, or at both positions [8]. The reported arabinoxylan content of commercial beers ranged from 790 to 1786 mg/L [5] and a considerable amount of it remained as arabinoxylan polymer. Arabinoxylan has been proven to form a highly viscous solution [3,4,5,6]. The effect of arabinoxylan on the viscosity and filtration rate of wort and beer is at least as important as that of β-glucan [3,4,5,7,9,10]. 

Supplementation of microbial xylanolytic enzymes to the mash could be a good choice for the degradation of arabinoxylan and has been demonstrated to improve the filterability of wort [11,12,13]. The microbial xylanolytic enzyme system includes mainly xylanase (endo-*β*-1,4-xylanase, EC 3.2.1.8), *α*-L-arabinofuranosidase (AFase) (EC 3.2.1.55), and *β*-D-xylosidase (EC 3.2.1.37) [14]. Xylanase is the major component of the xylanolytic enzyme system, which hydrolyses the linear β-1,4-D-xylopyranosyl backbone in the arabinoxylan. The action of xylanase drastically changes the structure and physicochemical properties of arabinoxylan. AFase is another major component of the xylanolytic enzyme system. AFase is a debranching enzyme. AFase catalyzes the hydrolysis of the arabinofuranosyl substituent from the arabinoxylan backbone, while β-D-xylosidase removes β-1,4-D-xylopyranosy moieties from the terminal end of arabinoxylo-oligosaccharides and plays a key role in the complete hydrolysis of arabinoxylan [15]. To our knowledge, there has been no investigation on the arabinoxylan degradation of barley malt by microbial AFases. 

The filamentous fungus *Trichoderma reesei (T. reesei)* is an excellent producer of xylanolytic enzymes [16,17]. Reports concerning AFases from *T. reesei* are quite limited. In previous work, we have found a complete xylanolytic enzyme system in the culture filtrate of *T. reesei* CICC 41,495 by secretome analysis [18]. This research focusses on the AFase isolated from that crude mixture of xylanolytic enzymes. Finally, a glycoside hydrolase (GH) family 62 AFase was purified to apparent homogeneity. The applicable potential of the purified AFase during Congress mashing with domestic barley malt was also evaluated. The results provide specific guidance on developing enzymatic strategies for arabinoxylan degradation during the mashing process.

## 2. Materials and Methods

### 2.1. Materials and Chemicals

*T. reesei* CICC 41,495 used in this study was purchased from the China Centre of Industrial Culture Collection (Beijing, China). Commercial malted barley (*Hordeum vulgare* L. cv. Dan’er, Chinese harvest 2017), with low β-glucan content (96 mg/L) and poor filtration efficiency, was obtained from a commercial malting company in the Jiangsu province of China.

Oat-spelt xylan, birchwood xylan, beechwood xylan, and 4-Nitrophenyl-*α*-L-arabinofuranoside (*p*-NPAF) were from Sigma-Aldrich (St Louis, MO, USA). Arabinoxylo-oligosaccharide was from Hualan Chemical (Shanghai, China). Soluble wheat arabinoxylan (medium viscosity) and insoluble wheat arabinoxylan were from Megazyme (Wicklow, Ireland). Sephadex G-25, DEAE-Sepharose Fast Flow and Sephacryl S-100 were from GE Healthcare (Uppsala, Sweden). The protein molar-mass marker was from Bio-Rad Laboratories (Shanghai, China). All other chemicals used were from Sinopharm Chemical Reagent Co. Ltd. (Shanghai, China), and were analytical grade. 

### 2.2. Enzyme Production

*T. reesei* CICC 41,495 was maintained on solid potato dextrose agar slant. For enzyme production, the inoculum was prepared in Erlenmeyer flask using Mandels’ medium [19]. A 10% (*v*/*v*) inoculum culture was added to 250-mL Erlenmeyer flasks containing 50 mL of Mandels’ medium supplemented with wheat bran at 1.0% (*w*/*v*). The flasks were incubated at 28 °C and 200 rpm for 168 h. The culture filtrate was harvested by centrifugation at 8000× *g* and 4 °C for 20 min and used for the purification of AFase.

### 2.3. Protein Concentration Estimation

Total protein concentration was determined using the Bradford method with bovine serum albumin (BSA) as a standard [20].

### 2.4. Enzyme Activity Assay

AFase activity throughout the purification procedure was determined using *p*-NPAF as the substrate (4 mM) according to the method of Oh et al. [21]. The substrate solution (250 μL) was mixed with 100 mM sodium acetate buffer (pH 5.5, 500 μL), and then a diluted enzyme solution (250 μL) was added. The mixture was incubated at 50 °C for 15 min. The reaction was stopped by the addition of 1.0 mL of 1.0 M Na_2_CO_3_ solution. The enzyme solution boiled in a water bath for 10 min was used as the negative control. The absorbance was measured at 420 nm and the amount of *p*-nitrophenol liberated was calculated from a standard curve. One unit of activity was defined as the amount of enzyme required to liberate one μmol *p*-nitrophenol from *p*-NPAF within one min under these conditions.

### 2.5. Enzyme Purification

To purify the AFase secreted by *T. reesei* CICC 41495, the crude enzyme in the culture filtrate was first fractionated by adding powdered (NH_4_)_2_SO_4_ to a 20–75% saturation. The precipitate was collected by centrifugation at 8000× *g* for 20 min (4 °C), dissolved in 20 mM Tris-HCl buffer (pH 8.0), and desalted on a Sephadex G-25 column (1.6 cm × 60 cm) with the same buffer. The desalted enzyme solution was loaded onto a DEAE-Sepharose Fast Flow column (1.6 cm × 20 cm) pre-equilibrated with 20 mM Tris-HCl buffer (pH 8.0). After washing off the unbound proteins with the starting buffer, the bound proteins were eluted by a linear gradient of NaCl (0−500 mM) in the same buffer at a flow rate of 100 mL/h. The AFase activity and the absorbance of each fraction at 280 nm (A_280_) were measured, respectively. Column fractions with AFase activity were pooled and further concentrated with a 3500 Da cut-off dialysis membrane embedded in PEG20000. The concentrated active fractions were further chromatographed on a Sephacryl S-100 column (1.6 cm × 95 cm) with 100 mM sodium acetate buffer (pH 5.5) containing 150 mM NaCl at a flow rate of 20 mL/h. The active fractions were pooled, lyophilized, and stored at −20 °C for further analysis.

### 2.6. Electrophoresis

The homogeneity and molar mass of the purified AFase were assessed by sodium dodecyl sulfate-polyacrylamide gel electrophoresis (SDS-PAGE) with a 5.0% (*w*/*v*) stacking gel and a 12.5% (*w*/*v*) separating gel using a Mini-PROTEAN^®^ 3 Cell system (Bio-Rad, USA) [22]. Proteins were stained with Coomassie Brilliant Blue G-250 and destained in a solution containing 50% (*v*/*v*) methanol and 10% (*v*/*v*) acetic acid overnight. A low molar mass protein marker (14.3–97.2 kDa, Takara, Dalian, China) was used as the standard.

### 2.7. Protein Identification 

The protein band with AFase activity was excised from the SDS-PAGE gel and digested with trypsin as described by Bienvenut et al. [23]. Spectra were acquired on Ultraflex matrix-assisted laser desorption/ionization two-stage time-of-flight tandem mass spectrometry (MALDI-TOF/TOF tandem mass spectrometry) (Bruker Daltonik, Bremen, Germany), then processed by Flexanalysis software and analyzed by Biotools software. An in-house Mascot server (http://www.matrixscience.com) was used for database searches.

### 2.8. Substrate Specificity

The substrate specificity of the purified AFase towards different arabinose-containing substrates was determined by measuring the reducing sugars in a reaction mixture. The reducing groups were measured by the 3,5-dinitrosalicylic acid (DNS) method [24]. The reaction mixture contained 500 μL of the purified AFase in 100 mM sodium acetate buffer (pH 5.5) and 500 μL of 1.0% (*w*/*v*) substrate solution in the same buffer. Substrates included oat-spelt xylan, birchwood xylan, beechwood xylan, soluble wheat arabinoxylan, insoluble wheat arabinoxylan, and arabinoxylo-oligosaccharide. The mixture was incubated at 50 °C for 30 min, followed by the addition of 1.25 mL DNS reagent to terminate the reaction. After mixing well, the reaction mixture was heated for exactly 5 min in a boiling water bath and the volume was made up to 6.25 mL with ultra-pure water. The catalytic activity was determined by analyzing the reducing sugars liberated from different substrates at 540 nm. The highest activity is defined as 100% and other activities are expressed as a percentage relative to the highest activity.

### 2.9. Hydrolytic Products Analysis

For hydrolytic products analysis, 1.0% (*w*/*v*) soluble or insoluble wheat arabinoxylan was digested at 50 °C for 16 h. The reaction mixture (1.0 mL) contained 500 μL of the purified AFase (5.0 μg) in 100 mM sodium acetate buffer (pH 5.5) and 500 μL of substrate solution in the same buffer. The reaction was terminated by boiling for 10 min. The released sugars in the reaction mixture were analyzed by Ion Chromatography (ISC5000, Thermo Fisher Scientific, USA) fitted with a pulsed amperometric detector and a CarboPac PA20 column (3 mm × 150 mm) with some modifications. The gradient elution was performed as described in the literature [25]. The flow rate was 0.50 mL/min. Arabinose and xylose were used as standards.

### 2.10. Mash Preparation with Supplemented AFase

The malt was first milled using a laboratory disc mill (Dezhijie, Beijing, China) at a fine grind setting of 0.2 mm. Wort was prepared according to the Congress mash method outlined in the Analytica-EBC [26]: Two hundred mL of water were mixed with 50 g of finely milled malt at 46 °C in stirred metal beakers. After continually stirring for 30 min at 45 °C, the temperature of the mash was increased to 70 °C at the rate of 1 °C/min for 25 min. More water (100 mL, 70 °C) was added. The temperature was maintained at 70 °C for 1 h. The mash was then cooled to 20 °C. The weight of the mash was adjusted to 450 g by the addition of water at 20 °C. To determine the effects of the purified AFase on the viscosity and filtration rate of the wort, a dosage series of the enzyme (0–20 mU/g malt) was added to the mash with Dan’er malt at the start of the mashing process.

At the end of the mashing, the filtration rate was determined, which was defined as the volume read after 30 min of filtration through fluted filter paper (the first 100 mL filtrate was returned to the funnel). The viscosity of wort was determined at 20 °C using a falling ball viscometer (Thermo Fisher Scientific, Germany). 

For the determination of the arabinoxylan polymer content of wort, absolute ethanol was added at a final concentration of 65% (*v*/*v*) [27]. Then, the mixture was shaken vigorously and kept at 4 °C overnight. The pellets were recovered by centrifugation for 20 min (8000× *g*, 4 °C) and redissolved in ultra-pure water. The arabinoxylan polymer content in the solution was carried out according to the phloroglucinol colorimetric method presented by Douglas [28], with some adaptations. Briefly, 0.1 mL of wort was mixed with 1.9 mL of ultra-pure water. Then 10 mL of the phloroglucinol reagent was added. The mixture was heated in a boiling water bath for 25 min. After cooling to room temperature, the volume in the test tube was adjusted to 25 mL with ultra-pure water. The arabinoxylan concentration was calculated by reading the absorbances at 510 and 552 nm and comparing them with the calibration curve, which was constructed with a stock solution of 100 mg/L D-(+)-xylose. 

## 3. Results and Discussion

### 3.1. Enzyme Purification

**Figure 1:**
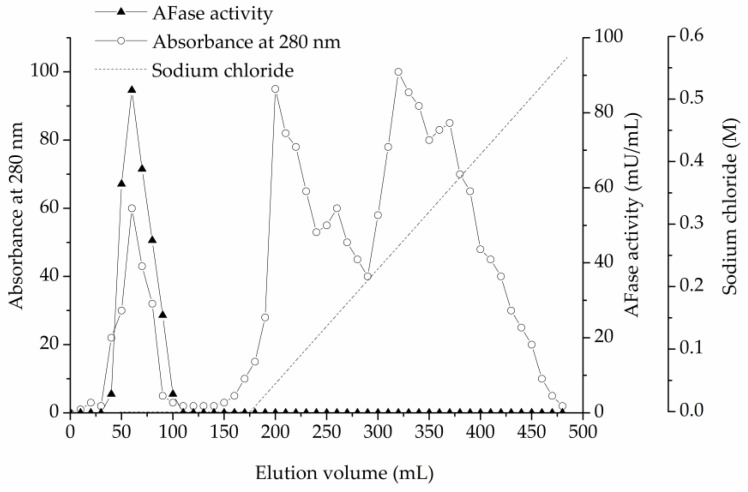
Ion-exchange chromatography of α-L-arabinofuranosidase (AFase) on diethylaminoethyl (DEAE) Sepharose Fast Flow.

**Figure 2:**
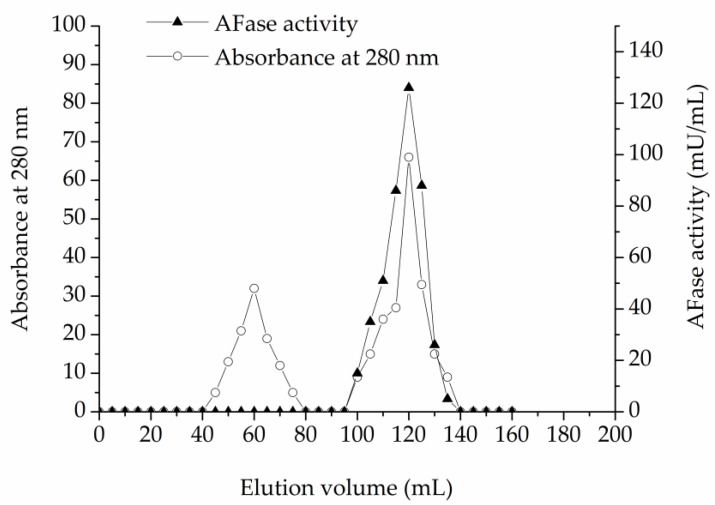
Gel-filtration chromatography of AFase on Sephacryl S-100.

As described in the materials and methods section, an AFase was purified to apparent electrophoretic homogeneity from the culture filtrate of *T. reesei* CICC 41495. Protein content and enzyme activity of each fraction were estimated at each step during the chromatography. The protein content was monitored at 280 nm. For direct UV measurement at 280 nm, the protein solution alone was used, without the addition of reagents, thus the measurements are quick. The AFase activity appeared as a major peak at the void volume through ion-exchange chromatography on the DEAE-Sepharose Fast Flow column, which is shown in Figure 1. Column fractions with AFase activity were pooled, concentrated, and further separated through gel-filtration chromatography on the Sephacryl S-100 column, and the elution profile is shown in Figure 2. 

**Figure 3:**
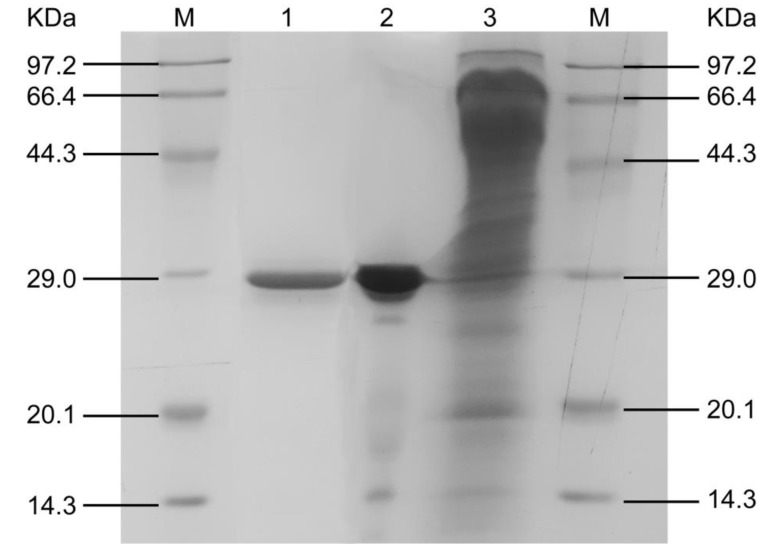
SDS-PAGE profiles during different stages of AFase purification. Lane 1, after Sephacryl S-100; lane 2, after DEAE-Sepharose Fast Flow; lane 3, culture filtrate; M, molar mass markers.

SDS-PAGE profile of each step during the purification is shown in Figure 3. The purified AFase appeared to be a single protein band with a molar mass of 29.0 kDa in the SDS-PAGE gel. 

The protein band with AFase activity was exercised and identified as a GH62 AFase (Accession number gi|589103163) (EC 3.2.1.55, termed as TrAbf62A in this paper) by MALDI-TOF/TOF tandem mass spectrometry (Table S1). The predicted molar mass and isoelectric point of the enzyme were 34.9 kDa and 6.4, respectively. 

The purification steps of TrAbf62A from the culture filtrate of *T. reesei* CICC 41,495 are summarized in Table S2. The overall purification fold of TrAbf62A was 11.2, and the yield was 26.5%.

According to the similarities of the amino acid sequence, AFases are classified mainly into families GH2, GH3, GH43, GH51, GH54, and GH62 [27]. The genome of *T. reesei* encodes five AFases (two belonging to GH43, two belonging to GH54, and one belonging to GH62) [29,30,31]. Poutanen [32] first reported an AFase purified from *T. reesei* by cation- and anion-exchange chromatography, which was an enzyme with a molar mass of 53 kDa, isoelectric point of 7.5, and pH optimum of 4.0. The enzyme preferred arabinoxylo-oligosaccharides produced by the endoxylanase and could release arabinose from wheat straw and beet arabinan, but had no endoxylanase activity. An AFase with a molar mass of 35 kDa has also been reported to be fractionated from *T. reesei*. Pepsin treatment showed that the 35 kDa AFase was a result from the proteolytic cleavage of the C-terminal region of the 53 kDa AFase [33]. However, the GH families of both AFases mentioned above are not clear. In this paper, only one AFase, which belongs to GH62, has been purified and identified, which is consistent with previous results [18]. The family GH62 exclusively contains AFases secreted by fungi or bacteria [15]. Family GH62 AFase is often not identified in the culture filtrate of *T. reesei*.

### 3.2. Substrate Specificity 

The substrate specificity of an enzyme is an important property for its specific uses. The substrate specificity of TrAbf62A towards arabinoxylan polymer and arabinoxylo-oligosaccharide is summarized in Table 1.

From the relative activities towards different arabinose-containing substrates stated in Table 1, TrAbf62A exhibited broad substrate specificity, but distinct differences were observed. TrAbf62A exhibited maximum hydrolytic activity towards arabinoxylo-oligosaccharide. TrAbf62A also showed considerable activity towards soluble wheat arabinoxylan, followed successively by oat-spelt xylan. TrAbf62A showed lower activity towards insoluble wheat arabinoxylan under the experimental conditions. The relative activity of TrAbf62A towards soluble wheat arabinoxylan was 11.2 times higher than that towards insoluble wheat arabinoxylan. No activity was detected with beechwood and birchwood xylans.

Although different arabinoxylans have the same linear backbone of the β-D-xylopyranosyl unit, their solubility, substitution degree, and substitution position are diverse. The diversity of structure and solubility between the arabinose-containing substrates used in this paper affects the relative activities of TrAbf62A. About 10.0–12.5% of the β-D-xylopyranosyl residues in oat-spelt xylan are mono-substituted by arabinofuranosyl residues at C-(*O*)-3 and di-substituted at the C-(*O*)-2 and C-(*O*)-3 positions [34]. Mono-substituted β-D-xylopyranosyl units account for 12–20% and di-substituted β-D-xylopyranosyl units take up 15–30% in soluble wheat arabinoxylan, which are much higher than that in oat-spelt xylan [35,36]. For insoluble wheat arabinoxylan, the substitution degree is higher than that in soluble wheat arabinoxylan [37], but a lower activity of TrAbf62A towards insoluble wheat arabinoxylan was observed. This can be explained by the less-soluble nature of insoluble wheat arabinoxylan, which requires additional enzymes to make the arabinofuranosyl moieties accessible for AFases. The results showed that birchwood xylan seemed resistant to the action of TrAbf62A. Birchwood xylan is also partly insoluble and has the simplest structure and contains trace numbers of substitution [38]. The structure and solubility of beechwood xylan are similar to birchwood xylan, the activities of TrAbf62A towards them had little difference.

The substrate specificity illustrated the difference of TrAbf62A activity on arabinoxylan polymer and arabinoxylo-oligosaccharide. Based on substrate specificity, AFases are classified into three groups [39]: Group A AFases preferentially act on *p*-NPAF and arabinoxylo-oligosaccharides, and are not active towards the branched arabinoxylan polymer; group B AFases are active on *p*-NPAF, arabinoxylo-oligosaccharides, and branched arabinoxylan polymer, while group C AFases specifically catalyze the hydrolysis of branched arabinoxylan, and are inactive on *p*-NPAF. The results above showed that TrAbf62A catalyzed the hydrolysis of *p*-NPAF, arabinoxylo-oligosaccharide, and arabinofuranosyl side-branched arabinoxylan. According to the above classification method, TrAbf62A was classified into group B.

### 3.3. Hydrolytic Products Analysis

**Figure 4:**
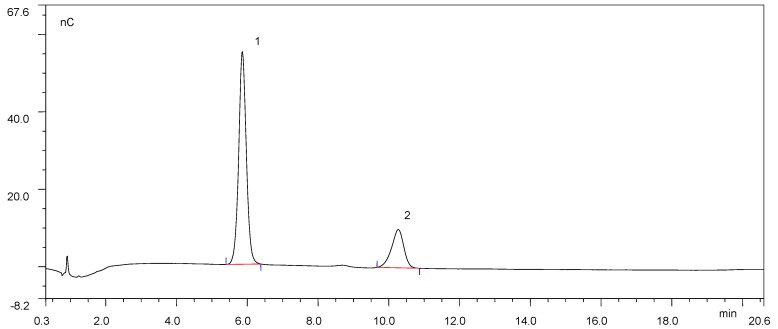
Chromatography profiles of the hydrolysates by TrAbf62A from soluble wheat arabinoxylan. 1, arabinose; 2, xylose.

The hydrolytic products in the enzyme–substrate mixture of TrAbf62A with soluble wheat arabinoxylan and insoluble wheat arabinoxylan were analyzed by ion chromatography (Figure 4 and Figure S1, respectively). 

According to the chromatogram (Figure 4), TrAbf62A released both arabinose (retention time 5.9 min) and xylose (retention time 10.317 min) from insoluble wheat arabinoxylan, accounting for 77.6% and 22.4% of the total sugars, respectively. On the contrary, there are no obvious peaks in the chromatogram (Figure S1). The chromatogram indicated that the sugar concentration in the reaction mixture with insoluble wheat arabinoxylan was low. The low sugar concentration was most likely due to the low activity of TrAbf62A on insoluble wheat arabinoxylan, which was consistent with the results in Table 1.

The sugar profile confirmed that TrAbf62A could cleave the arabinofuranosyl residues from β-D-xylopyranosyl units in branched arabinoxylan. GH62 AFases have been reported to have the capacity of liberating arabinose from arabinoxylan polymers [40,41,42,43]. Surprisingly, the chromatography profile also shows that TrAbf62A exhibited exoxylanase activity and released xylose from soluble wheat arabinoxylan. Several AFases are reported to show multifunctional activities towards arabinose-containing substrates. An AFase from radish seed (*Raphanus sativus* L.) has been reported to attack soluble wheat arabinoxylan in an exocleaving manner to release xylose [44]. A GH51 AFase from *Alicyclobacillus* sp. has also been reported to exhibit exoxylanase activity on soluble wheat arabinoxylan and sugar beet arabinan [45]. Wood and Macrae [46] reported that a GH62 AFase from *Aspergillus awamori* also showed the capacity to split off β-D-xylopyranosyl units from the main chain of arabinoxylan. A dual-function AFase (ARA-I) is also present in malted barley [47]. ARA-I hydrolyzes arabinoxylans at a low rate but plays an important role in the complete depolymerization of arabinoxylans through its ability to hydrolyze arabinoxylo-oligosaccharides. This implicated that there is an endogenous enzyme with similar function to TrAbf62A in the mash. The results provided more details on the mode of action of TrAbf62A. TrAbf62A showed potential for the degradation of soluble arabinoxylan in industrial biotechnology.

### 3.4. Supplementation of TrAbf62A during Mashing with Barley Malt

The efficient degradation of soluble arabinoxylan polymer in the mash during the beer brewing process is highly desirable for lautering improvement.

The Dan’er barley (*Hordeum vulgare* L. cv. Dan’er) is a widely cultivated variety in the Jiangsu Province of China. The characteristics of its commercial malt are listed in Table 2.

For most commercial malts used for beer production in China, the filtration rate of 150 mL/30 min is low, and 180–200 mL/30 min is the expected value. The data in Table 2 show that the filtration efficiency of Dan’er commercial malt was poor, with low β-glucan and high arabinoxylan polymer content. This is different from the usual reason that the filtration deficiency of commercial malt is due to low Kolbach index (KI) and high β-glucan content. Maltsters and brewers have been plagued by this for years, and the commercial prospects of this domestic malt were poor; hence, TrAbf62A was supplemented during the mashing process to try to solve this problem.

**Figure 5:**
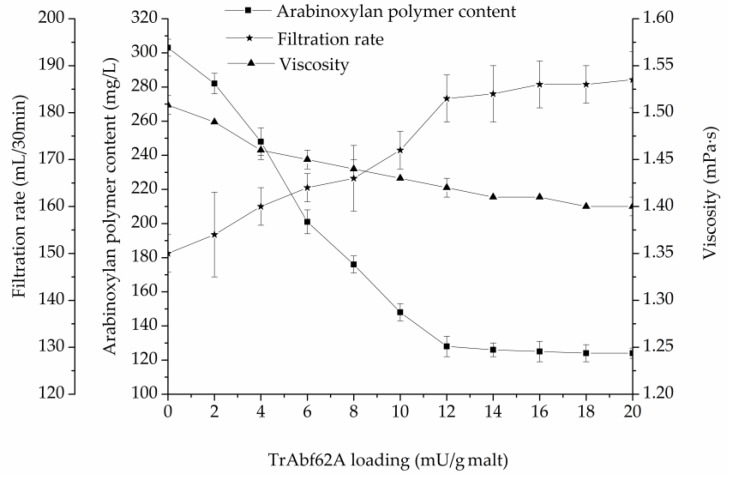
Effects of supplemented TrAbf62A on the arabinoxylan polymer content, viscosity, and filtration rate of wort.

The effects of TrAbf62A supplementation on the arabinoxylan polymer content, viscosity, and filtration rate during mashing are illustrated in Figure 5.

The results in Figure 5 show that TrAbf62A supplementation improved the lautering performance of the mash. When TrAbf62A was supplemented at a dosage of 12 mU/g of malt, the arabinoxylan polymer content of wort decreased by 36.3%, compared with the control, from 303 mg/L to 128 mg/L. At the same time, the viscosity reduced by 5.6%, from 1.51 mPa·s to 1.42 mPa·s, and the filtration rate improved by 22.1%, from 150 mL/30 min to 183 mL/30 min. These indices changed little with the further increase of the TrAbf62A dosage. TrAbf62A shows an applicable potential in improving the lautering performance of barley malt during mashing. The Dan’er barley used in this paper is a widely cultivated variety in the Jiangsu Province of China. However, the high content of arabinoxylan in this variety causes filtration deficiency and makes it unusable. Supplementation of TrAbf62A increased the filtration rate of the Dan’er malt to near normal levels. This study provides a solution to overcome the filterability deficiency of malted barley. The malted barley can be used to replace a certain proportion of imported malted barley for beer production.

Arabinoxylan polymers precipitated with 65% ethanol from barley malt constituted of 5.1–7.1% mono-substituted β-D-xylopyranosyl units by arabinofuranosyl residues at the C-(*O*)-3 position, 5.1–6.8% mono-substituted at the C-(*O*)-2 position, and 24.8–28.0% di-substituted at both C-(*O*)-2 and C-(*O*)-3 positions to the same β-D-xylopyranosyl unit [48]. This may be the reason why the supplementation of TrAbf62A improved the lautering performance of the mash. The arabinofuranosyl residues may sterically hinder the access of some xylanases to the backbone of arabinoxylans [49,50]. As debranching enzymes, AFases remove the arabinofuranosyl residues linked to the β-1,4-D-xylopyranosyl units to make xylanase more accessible to attack arabinoxylans, thus promote the degradation of arabinoxylan polymers.

## 4. Conclusions

TrAbf62A was most active against soluble, highly substituted arabinoxylan. Hydrolytic product analysis showed that TrAbf62A not only has the function of removing arabinofuranosyl residues from β-D-xylopyranosyl units, but also exhibited exoxylanase activity towards soluble wheat arabinoxylan. Supplementation of TrAbf62A during mashing decreased the arabinoxylan polymer content of wort, and as a consequence, showed performance in viscosity reduction and filtration rate improvement. TrAbf62A has the prominent applicable potential for lautering performance improvement during mashing.

## Figures and Tables

**Table 1:** Substrate specificity of TrAbf62.

Substrate (1.0%, *w*/*v*)	Relative Activity (%) ^1^
arabinoxylo-oligosaccharide	100
Oat-spelt xylan	44.1 ± 0.3
Soluble wheat arabinoxylan	87.5 ± 0.6
Insoluble wheat arabinoxylan	7.8 ± 0.05
Birchwood xylan	0
Beechwood xylan	0

**^1^** Values represent the mean ± SD (*n* = 3). The highest activity is defined as 100%, and the others are expressed as a relative value to the highest activity.

**Table 2:** Characteristics of the commercial malted barley used in this manuscript.

Variable	Value
Moisture (%)	4.4 ± 0.2
Color (EBC)	4.33 ± 0.14
Turbidity (EBC)	1.36 ± 0.03
Free amino nitrogen (mg/L)	182 ± 2
Extract (%)	79.1 ± 0.1
Total protein (%)	12.85 ± 0.21
Kolbach index (%)	44.6 ± 0.4
β-glucan (mg/L)	96 ± 3
Filtration rate (mL/30min)	150 ± 7
Arabinoxylan polymer content (mg/L)	303 ± 5
Friability (%)	74.2 ± 1.2
Viscosity (mPa·s)	1.51 ± 0.01

## Supplementary Materials

The following are available online at https://www.mdpi.com/2304-8158/9/3/356/s1, Figure S1: Chromatography profiles of standards and the hydrolysates by TrAbf62A from soluble and insoluble wheat arabinoxylans, Table S1: Information of the protein band with AFase activity, Table S2: Purification summary of TrAbf62.
