# Supplementary material for: A Glycoside Hydrolase Family 62 A-L-Arabinofuranosidase from Trichoderma Reesei and Its Applicable Potential during Mashing
Source: Foods. 2020 Mar 19;9(3):356. doi: 10.3390/foods9030356 (PMC7143738; doi:10.3390/foods9030356)
Supplement: Supplementary file 1 [file foods-09-00356-s001.pdf]

Supplementary materials

Table S1. Information of the protein band with AFase activity.

Item	Result
Protein name	α-L-arabinofuranosidase
Accession number	gi丨589103163
CAZy family	GH 62
Mascot score	326
Predicted molar mass/ isoelectric point [1]	34.9/6.4

[1] The predicted molar mass and isoelectric point were acquired from ProtParam tool (https://web.expasy.org/protparam/).

Table S2. Purification summary of TrAbf62.

Purification Step	Total Protein (mg)	Total Activity (mU) [1]	Specific Activity (mU/mg)	Purification Fold	Yield (%)
Culture filtrate	280	20720	74	1	100
$(NH_4)_2SO_4$ fractionation	123	15483	126	1.7	74.7
Sephadex G-25	108	12308	133	1.8	59.4
DEAE-Sepharose Fast Flow	13.5	7092	525	7.1	34.2
Sephacryl S-100	6.6	5492	832	11.2	26.5

[1] The activity was assayed at pH 5.5.

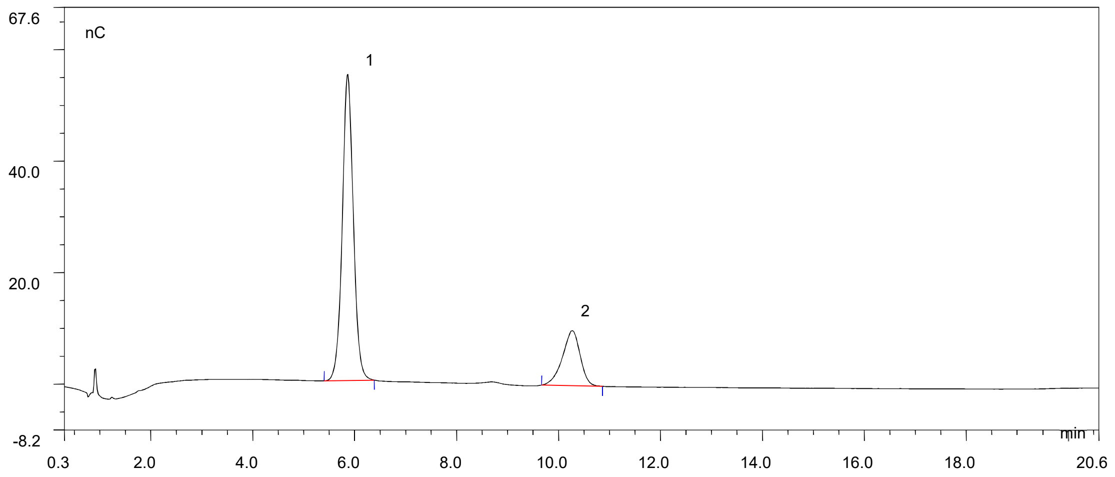

(a) soluble wheat arabinoxylan

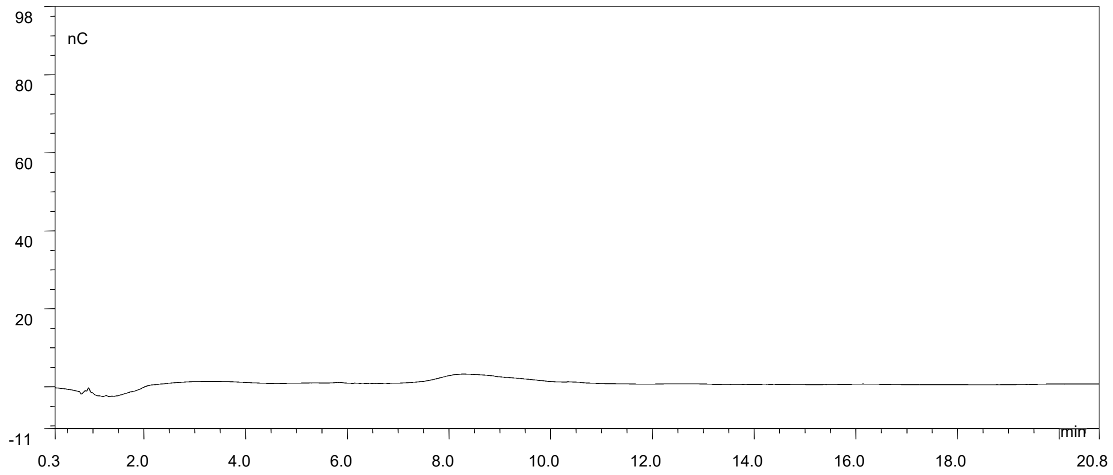

(b) insoluble wheat arabinoxylan

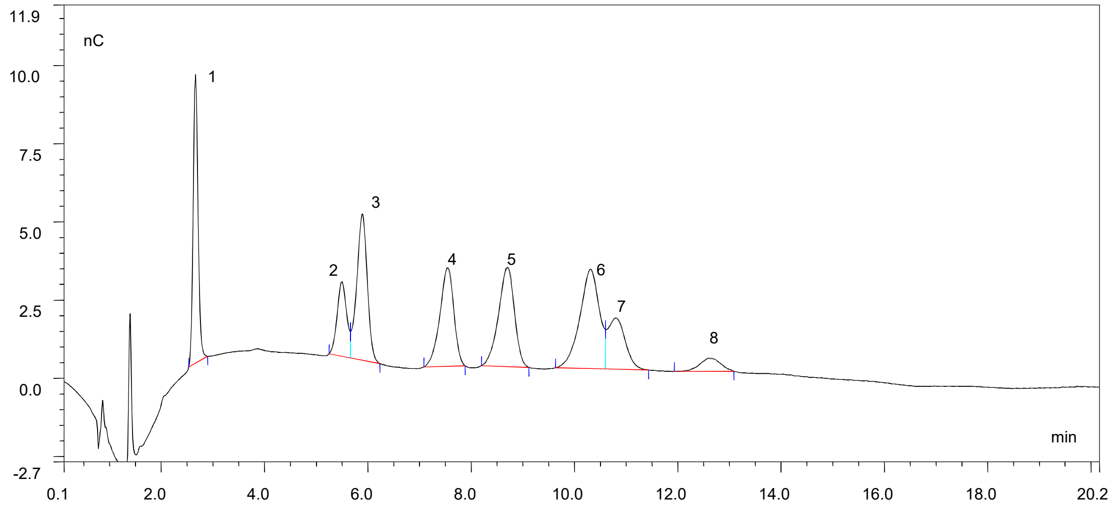

(c) standard sugars

No.	Sugar Name	Retention Time (min)	Concentration (mg/L)
1	fucose	2.667	5
2	rhamnose	5.5	5
3	arabinose	5.9	5
4	galactose	7.55	5
5	glucose	8.7	5
6	xylose	10.317	5
7	mannose	10.8	5
8	fructose	12.617	5

Figure S1 Chromatography profiles of standards (**c**) and the hydrolysates by TrAbf62A from soluble (**a**) and insoluble (**b**) wheat arabinoxylans.
